# Supplementary figures and images for: Alternative splicing downstream of EMT enhances phenotypic plasticity and malignant behavior in colon cancer
Source: eLife. 2022 Nov 8;11:e82006. doi: 10.7554/eLife.82006 (PMC9674345; doi:10.7554/eLife.82006)

Figure 1C

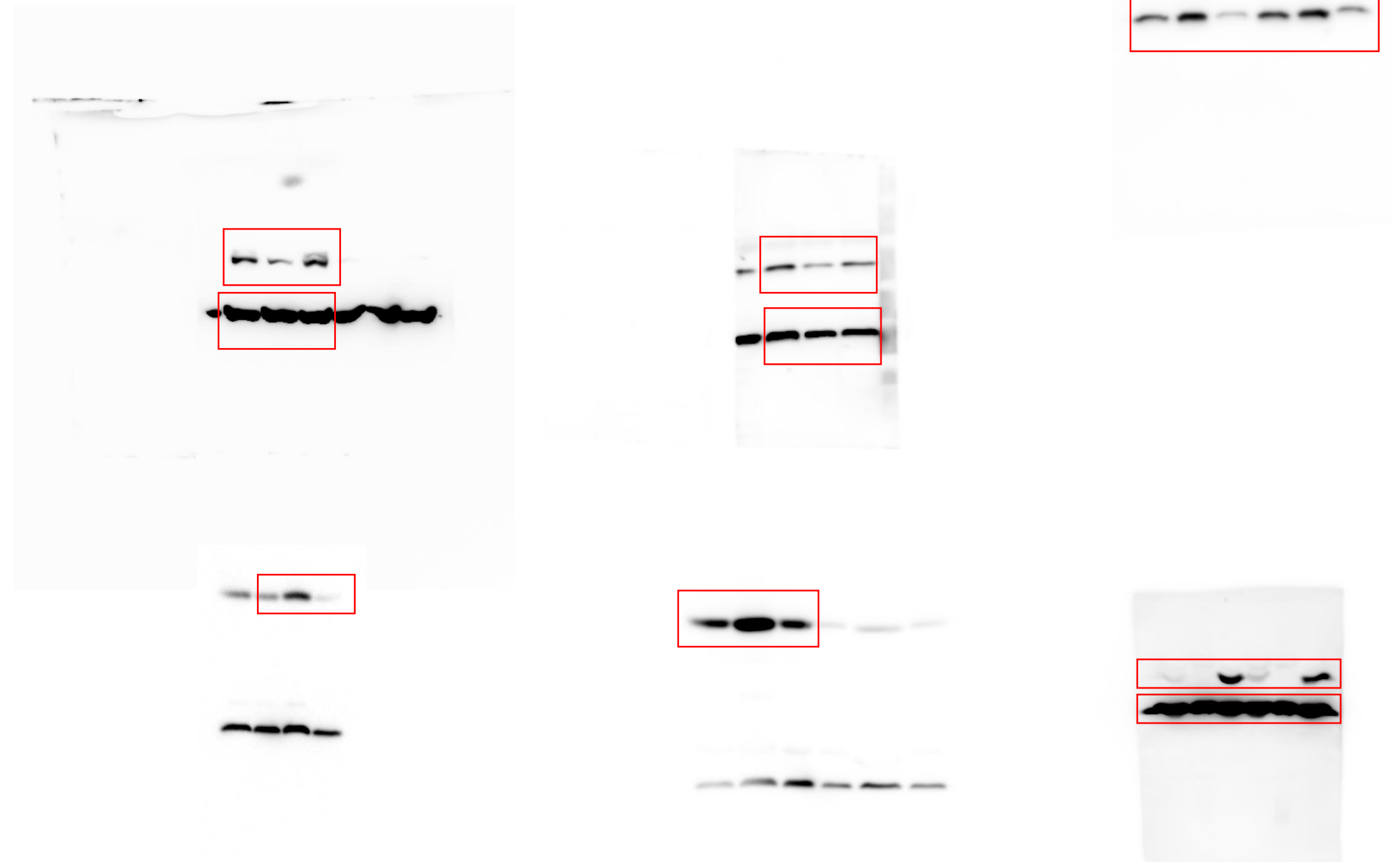

Supplement: Figure 1—source data 1. [file elife-82006-fig1-data1.pdf]

Figure 2D

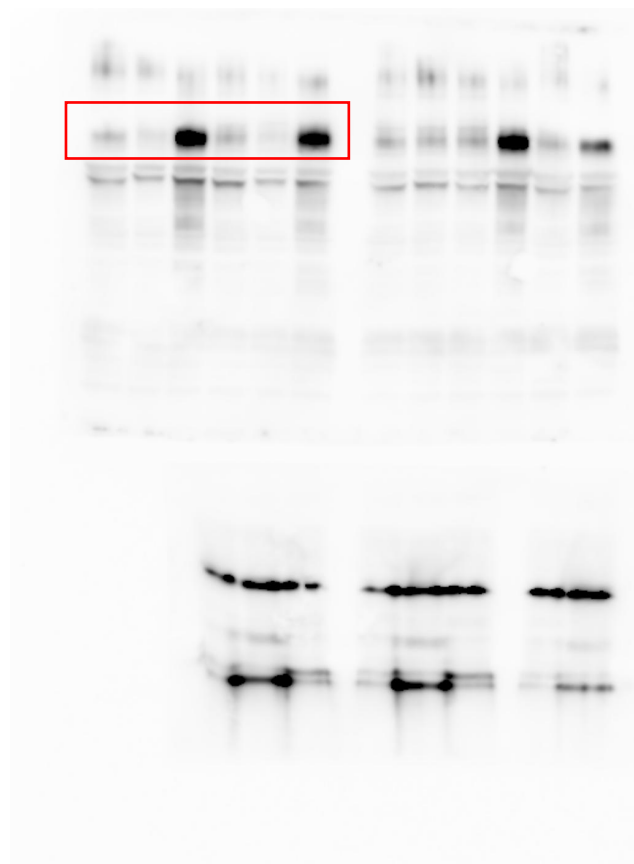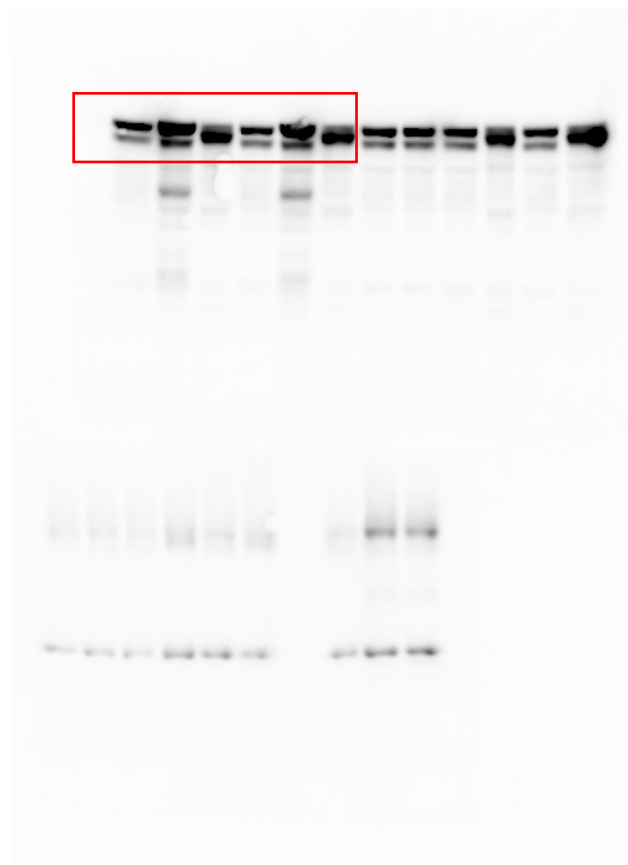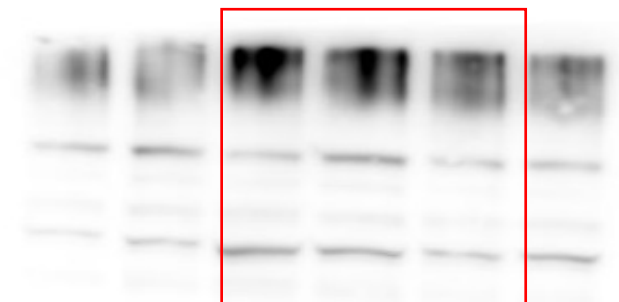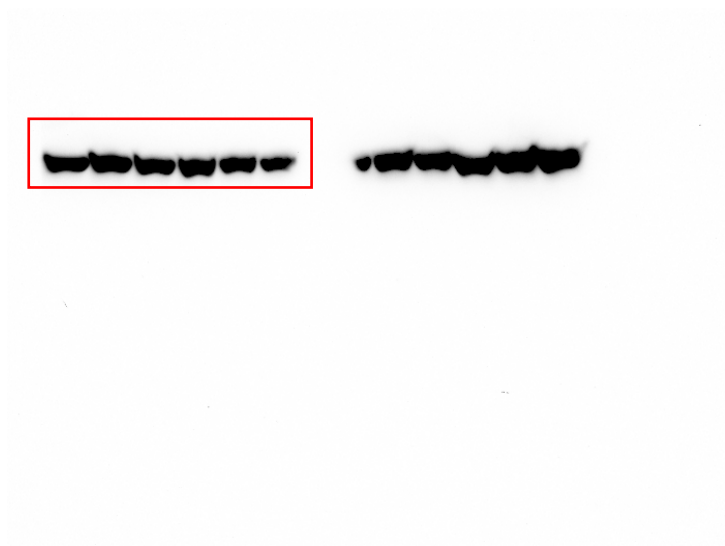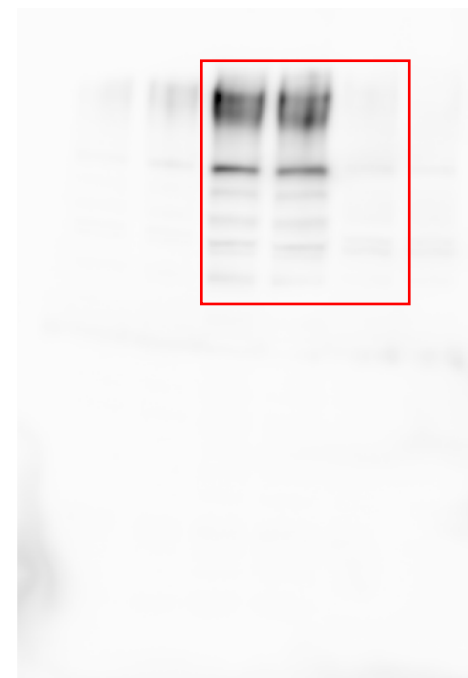

Supplement: Figure 2—source data 1. [file elife-82006-fig2-data1.pdf]

Figure 3A

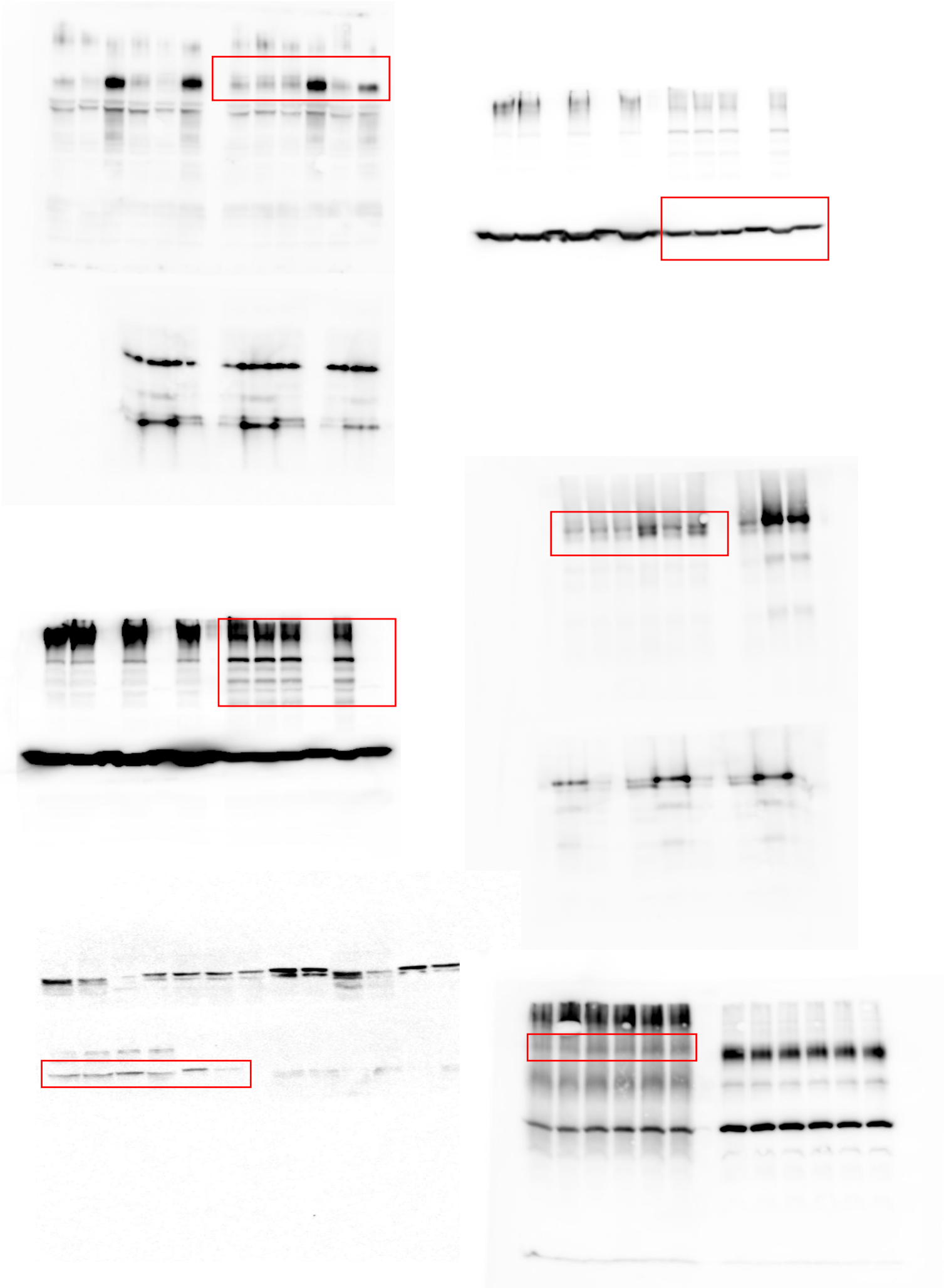

Supplement: Figure 3—source data 1. [file elife-82006-fig3-data1.pdf]

Figure 3B

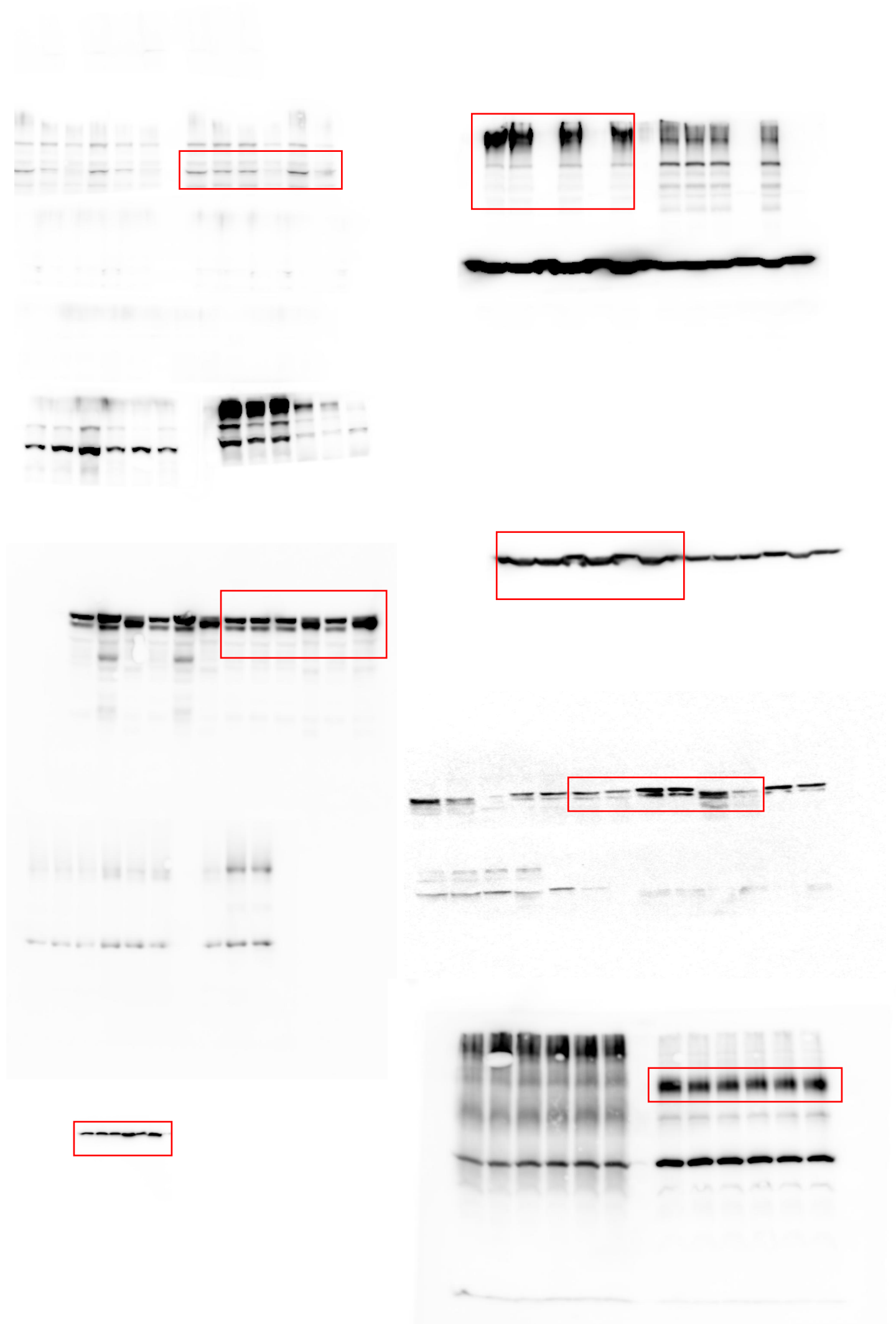

Supplement: Figure 3—source data 2. [file elife-82006-fig3-data2.pdf]

Figure 3C

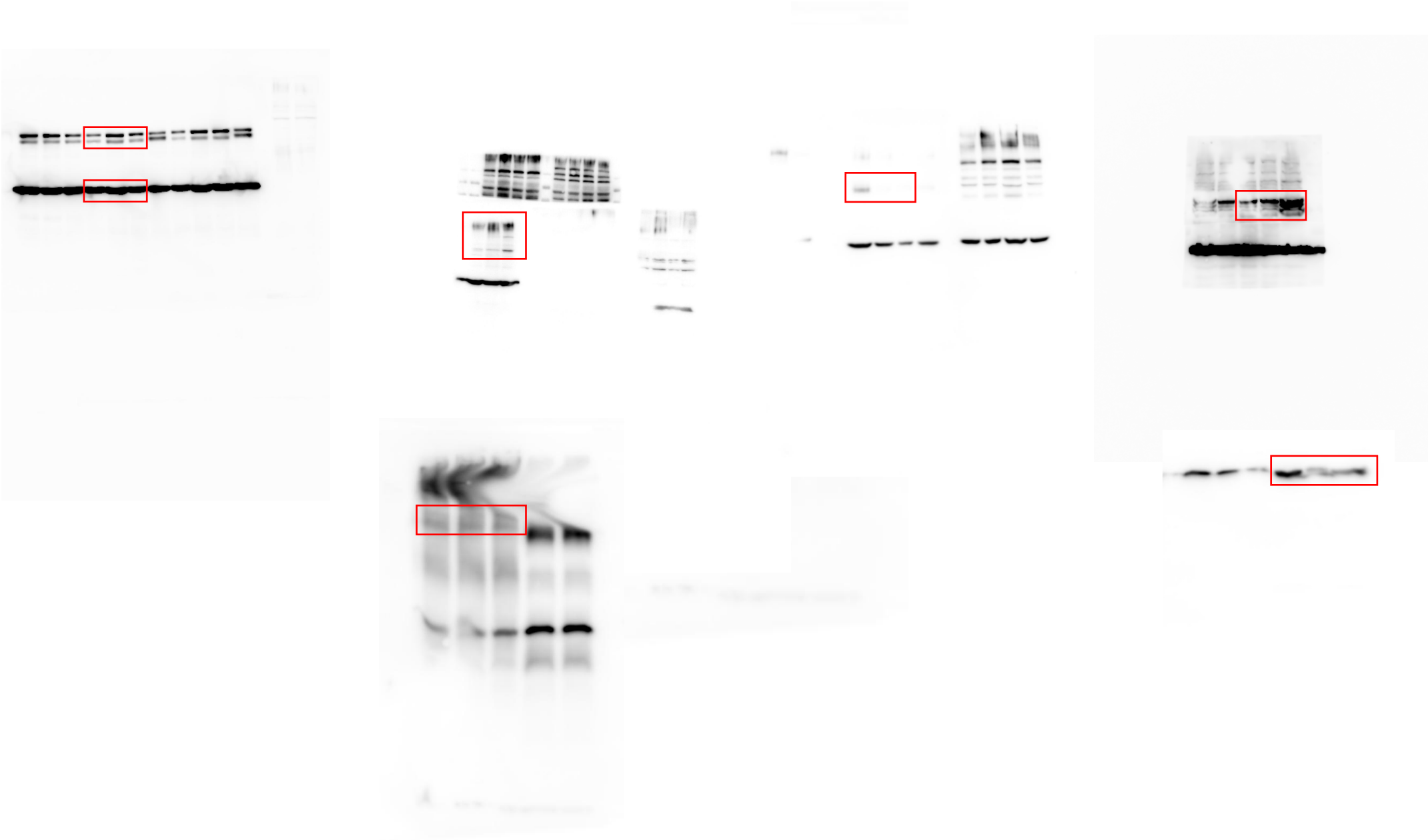

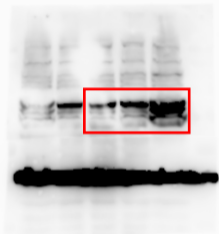

Supplement: Figure 3—source data 3. [file elife-82006-fig3-data3.pdf]

Figure 3D

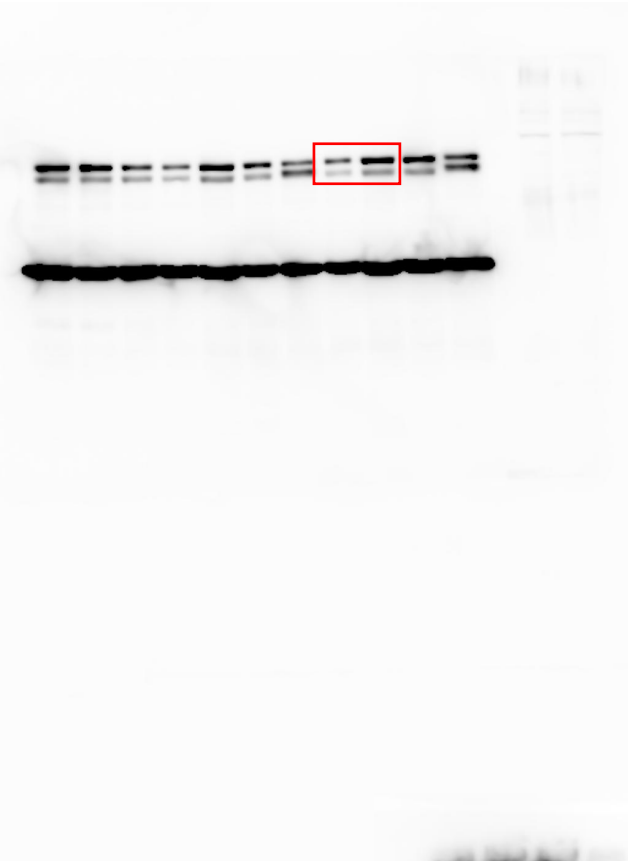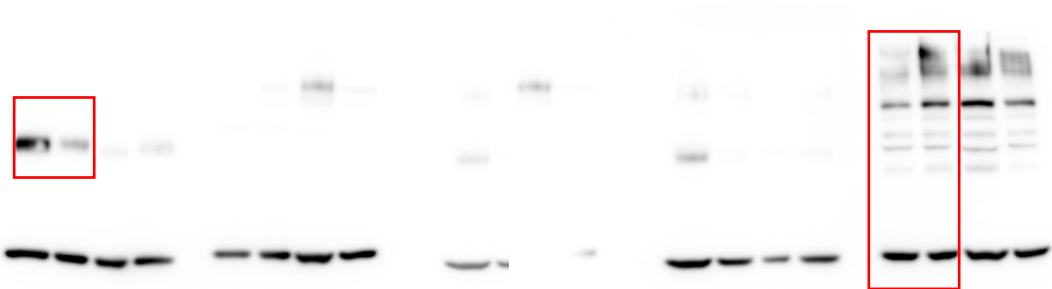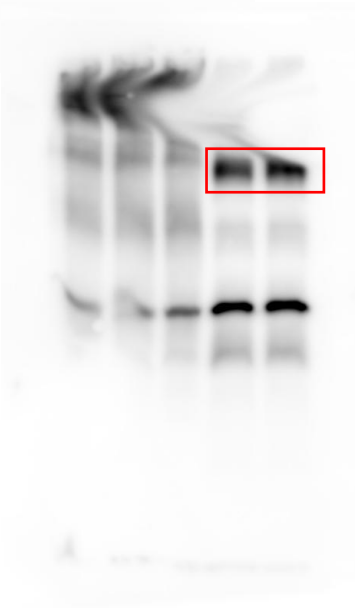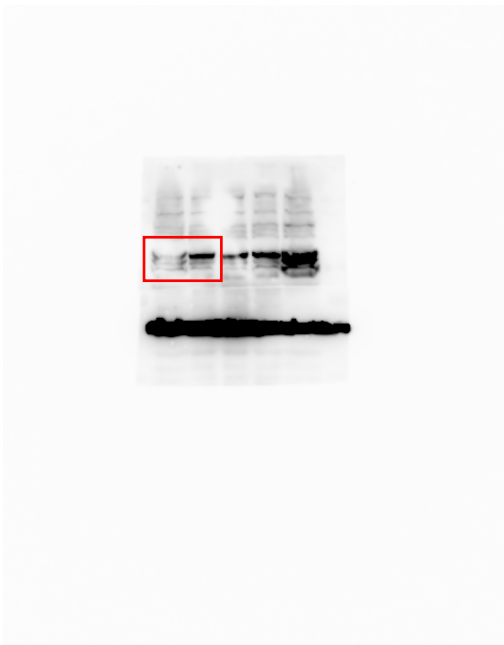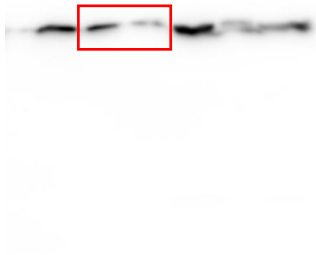

Supplement: Figure 3—source data 4. [file elife-82006-fig3-data4.pdf]

Suppl Figure 3A

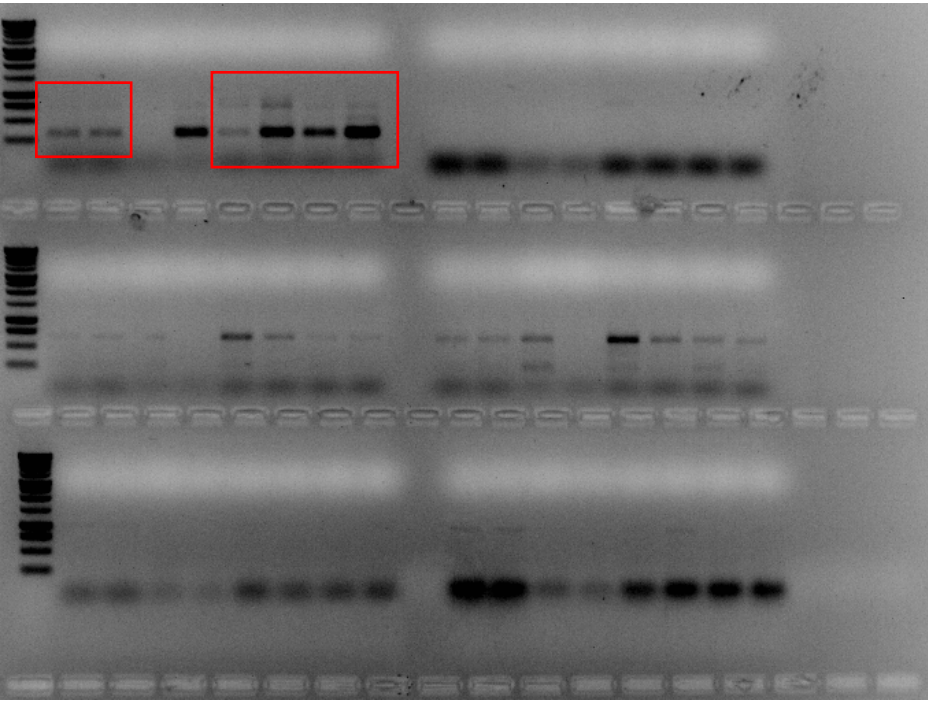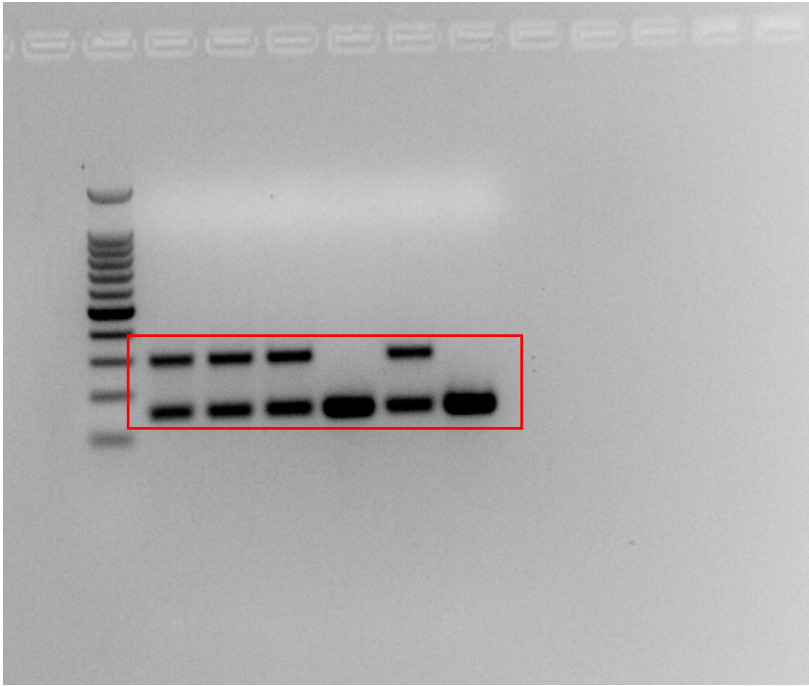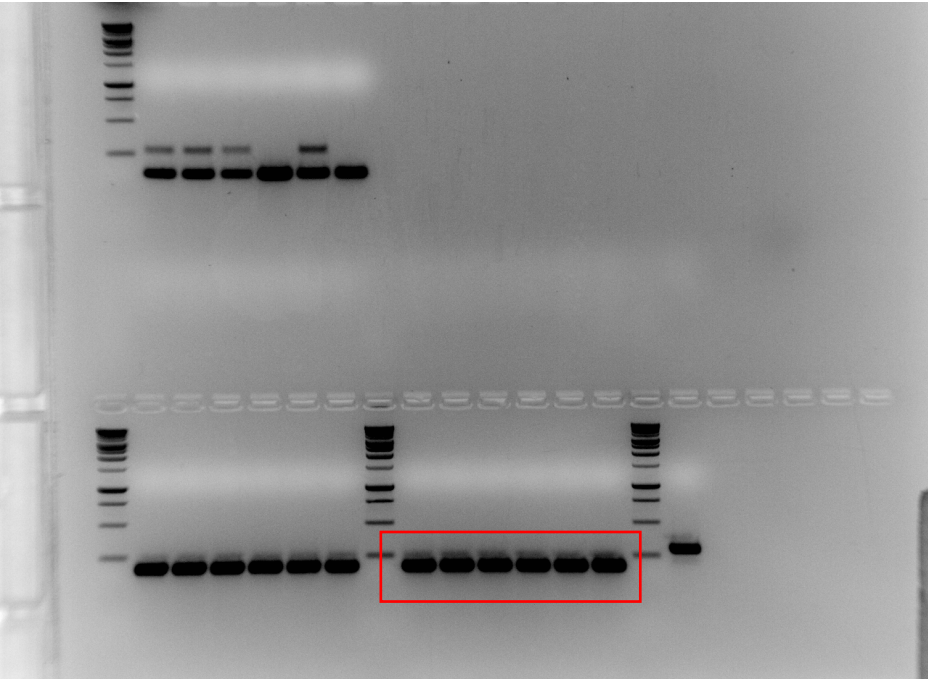

Supplement: Figure 3—figure supplement 1—source data 1. [file elife-82006-fig3-figsupp1-data1.pdf]

Suppl Figure 3B

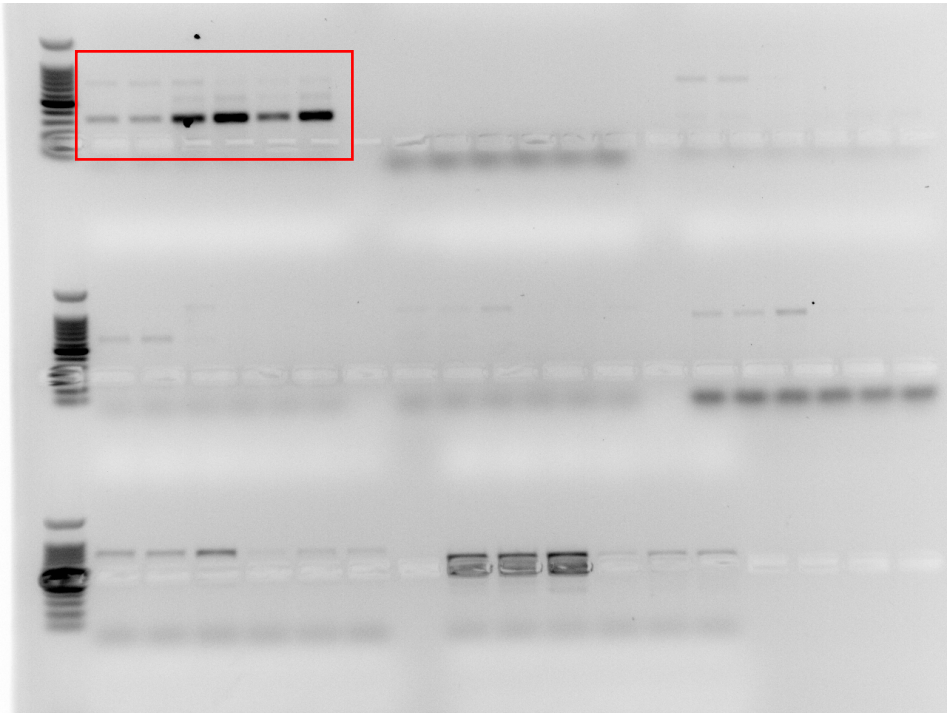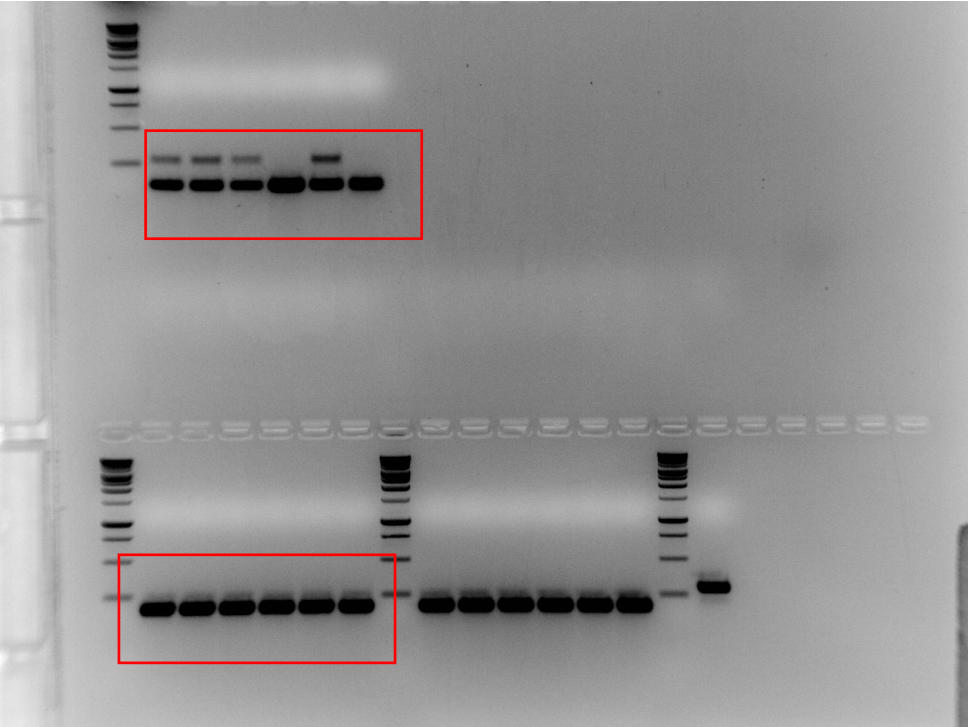

Supplement: Figure 3—figure supplement 1—source data 2. [file elife-82006-fig3-figsupp1-data2.pdf]

Suppl Figure 3C

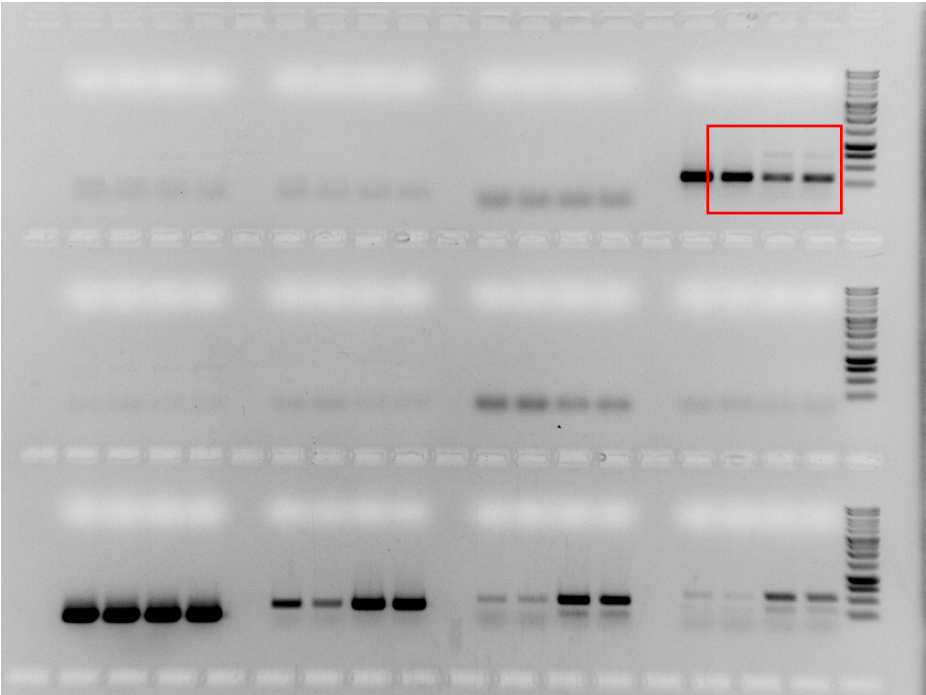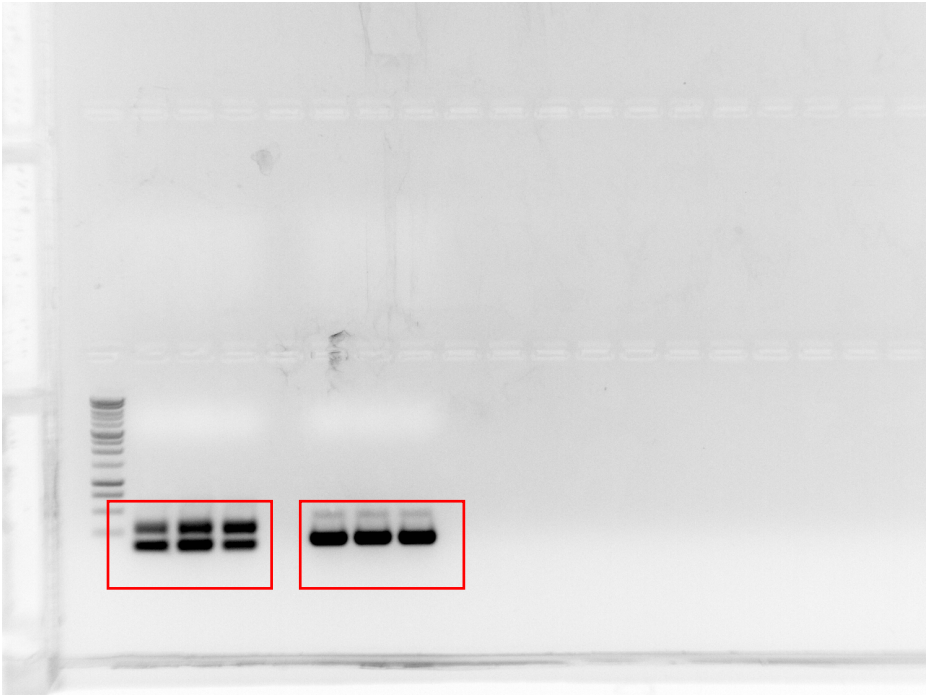

Supplement: Figure 3—figure supplement 1—source data 3. [file elife-82006-fig3-figsupp1-data3.pdf]

Suppl Figure 3D

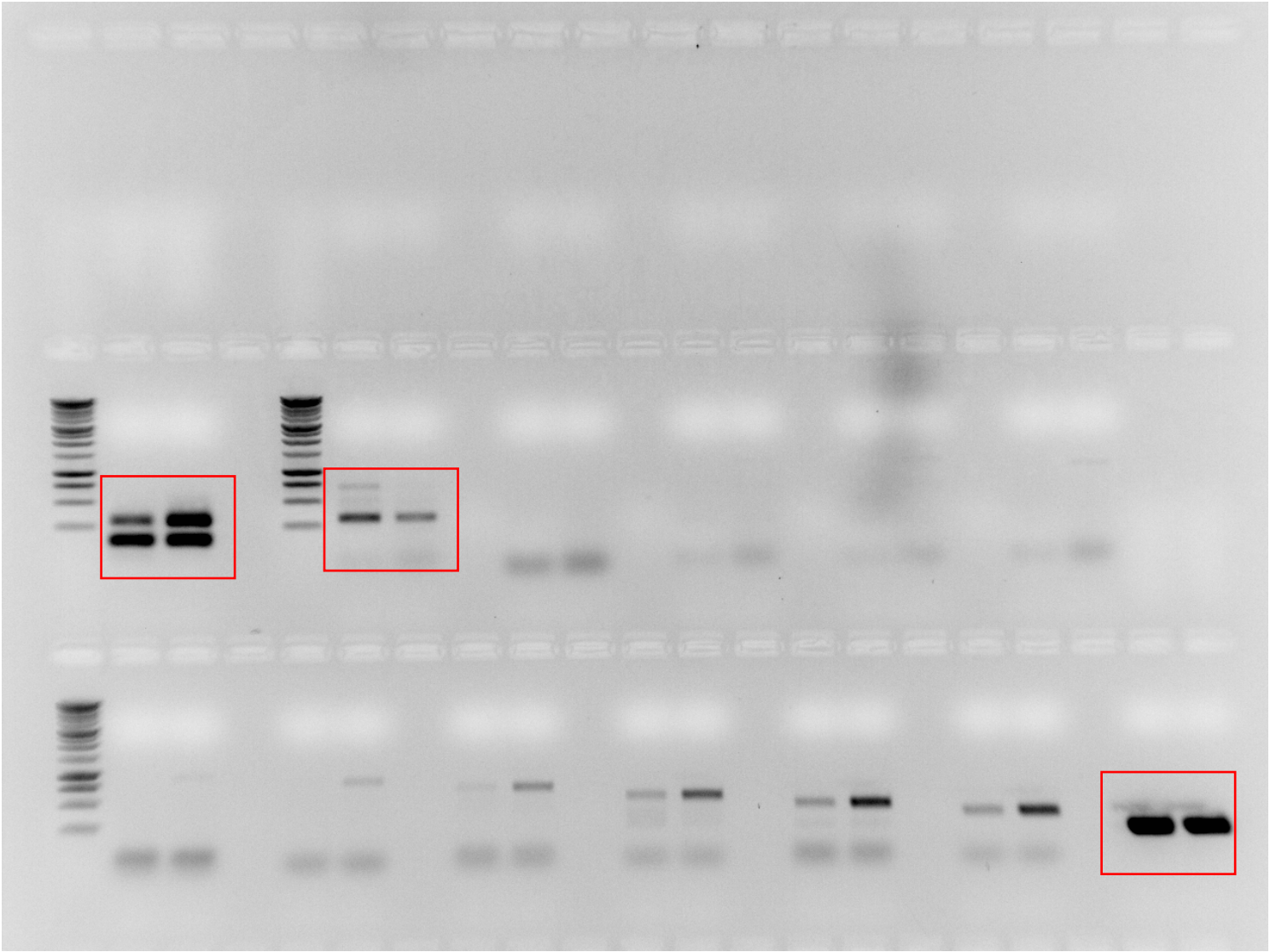

Supplement: Figure 3—figure supplement 1—source data 4. [file elife-82006-fig3-figsupp1-data4.pdf]

Suppl Figure 3F

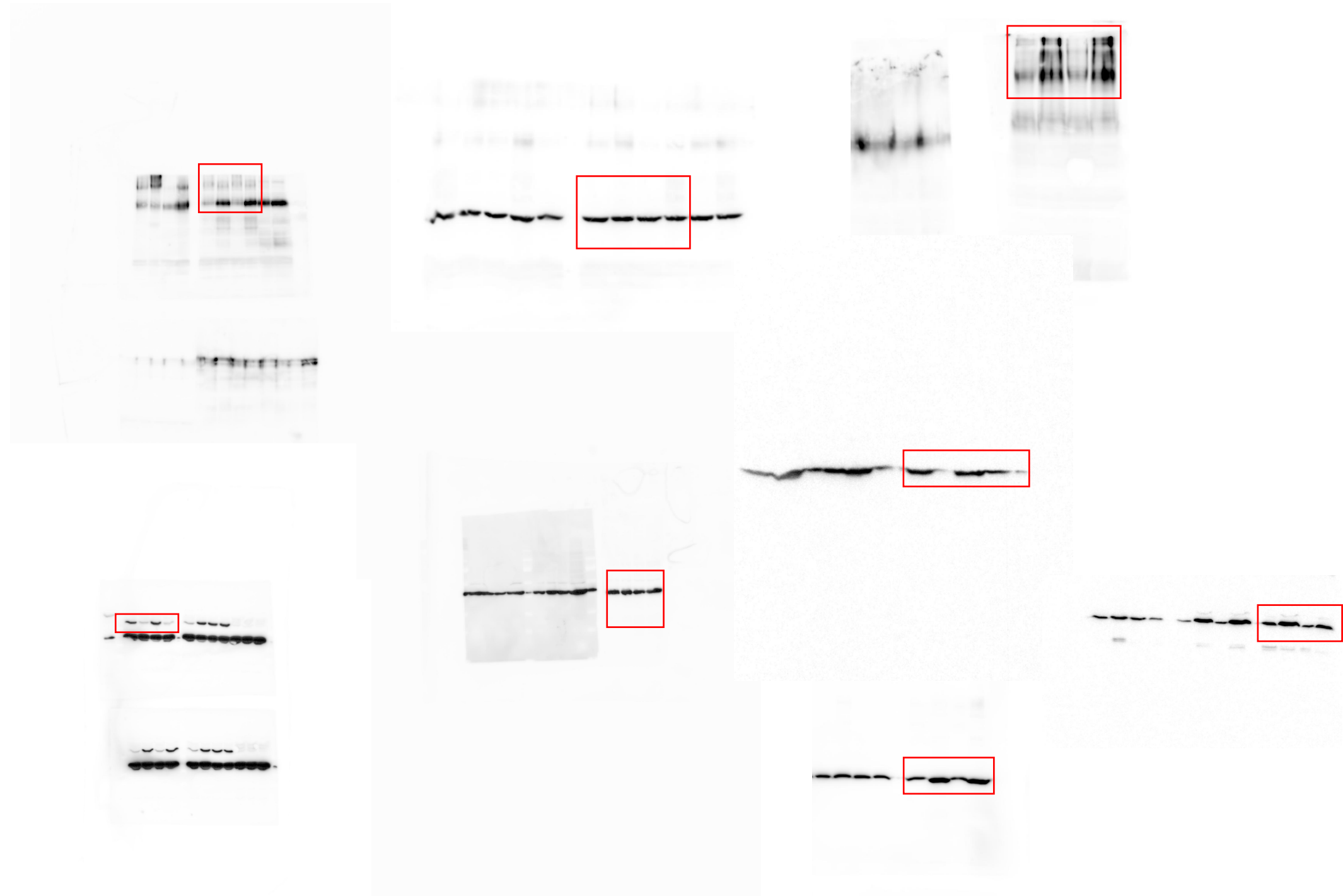

Supplement: Figure 3—figure supplement 1—source data 5. [file elife-82006-fig3-figsupp1-data5.pdf]

Suppl Figure 3H

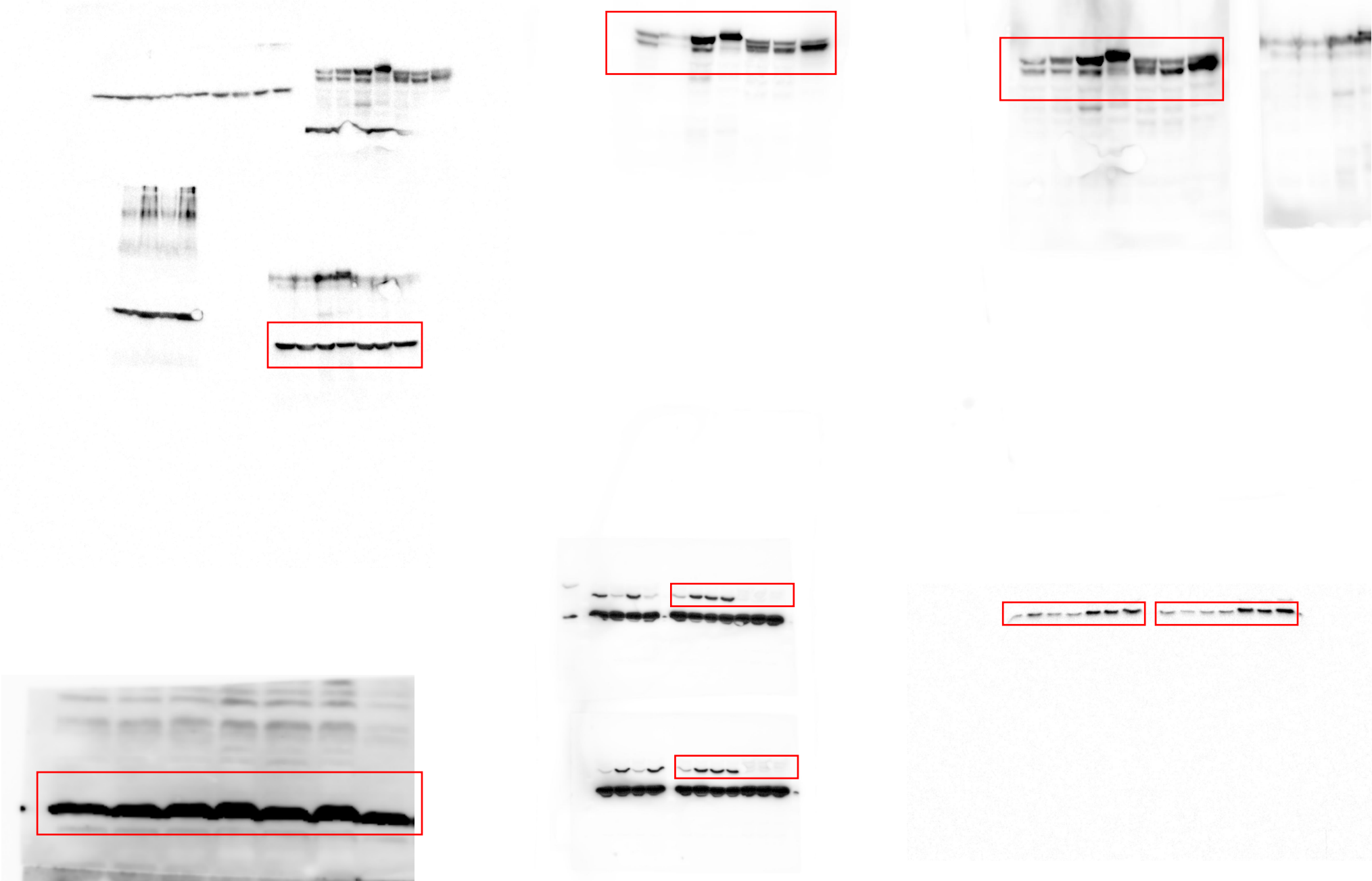

Supplement: Figure 3—figure supplement 1—source data 6. [file elife-82006-fig3-figsupp1-data6.pdf]
